# Recognition, Stabilization, and Command-Mapping Algorithms for Contactless Hand-Gesture Control of an ABB Industrial Robot

**DOI:** 10.3390/s26217073

**Published:** 2026-11-09

**Authors:** Bartosz Bieńkowski, Wojciech Kaczmarek

**Affiliations:** 1 Faculty of Electronics, Military University of Technology, 00-908 Warsaw, Poland; bartosz.bienkowski@proton.me; 2 Faculty of Mechatronics, Armaments, and Aerospace, Military University of Technology, 00-908 Warsaw, Poland

**Keywords:** hand gesture recognition, vision-based sensing, human–robot interaction, contactless robot control, cellular automaton, command mapping, industrial robot

## Abstract

**Highlights:**

**What are the main findings?**

- A lightweight hand-gesture control framework was developed that combines MediaPipe landmark normalization, MLP classification, and a cellular-automaton-based transition stabilizer.
- The proposed two-hand command grammar was successfully validated for robot connection, RAPID program control, and Cartesian motion control of an ABB IRB 120 robot in both virtual and physical environments.
- Temporal stabilization improved command reliability by reducing the impact of transient gesture-recognition errors before command execution.

**What are the implications of the main findings?**

- The framework provides a practical perception-to-control interface for contactless industrial robot operation.
- Separating gesture recognition, temporal validation, and command mapping reduces the likelihood of unintended robot actions caused by unstable classifier outputs.
- The architecture is extensible and can be adapted to additional gestures, robot functions, and future safety-oriented validation mechanisms.

**Abstract:**

Although contactless hand-gesture interfaces can support intuitive human–robot interaction, transient classification errors may trigger unintended commands when classifier outputs are mapped directly to robot actions. This study presents a processing framework for contactless control of an ABB IRB 120 industrial robot. The framework combines RGB acquisition, geometric normalization of 21 MediaPipe hand landmarks, population-based sample filtering, multilayer perceptron classification, temporal transition stabilization, and a two-hand command grammar. Thirteen gesture classes were represented by 63-dimensional landmark vectors, and 19,250 samples were retained after processing. The classifier achieved an approximate mean per-class recall of 99.46% on the held-out test subset. Four temporal processing configurations were compared using 30 repetitions per interface action: raw output, majority voting, three-consecutive-label confirmation, and the proposed cellular-automaton (CA)-based stabilizer. The latter two methods improved command reliability relative to the raw output and produced comparable results. The CA stabilizer processed labels sampled every 100 ms and introduced a validation delay of approximately 300 ms. Robot-program control achieved an 86.7% success rate across 90 trials, while Cartesian direction control achieved a 97.2% success rate across 180 trials. The mean displacement overshoot was 2.42 mm, or 24.2% of the nominal 10 mm command. The framework provides a practical perception-to-control interface while complementing, rather than replacing, certified robot safety functions.

## 1. Introduction

Industrial human–robot interaction is increasingly moving beyond operator panels and expert programming toward interfaces that allow operators to communicate their intentions more naturally. Contactless hand-gesture control is particularly appealing because it reduces dependence on physical input devices and can support rapid intervention while the operator remains focused on the workstation. However, in an industrial setting, a recognized gesture is not always a reliable control event. Occlusion, image disturbances, and hand-shape changes during transitions can generate short-lived erroneous labels that should not be mapped directly to robot actions.

This study developed and evaluated a vision-based hand-gesture interface for an ABB IRB 120 industrial robot, extending earlier work in which gestures and voice commands were used to control the same robot in virtual and physical environments [1]. Here, the interaction channel was limited to hand gestures, and the processing chain was expanded to include geometric landmark normalization, population-based training data filtering, lightweight classification, temporal transition stabilization, and context-dependent command interpretation. The objective was to transform a time-varying stream of classifier labels into robust control events before they reach the robot execution layer.

### Related Work

Research on robot operator interfaces encompasses conventional HMIs, alternative physical devices, and natural interaction modalities. A graphics tablet has been used to create and modify robot trajectories without direct RAPID programming [2], while an operator panel for an ABB GoFa robot and its track provided explicit mode selection and deterministic communication via a PLC and the PROFINET protocol [3]. Vision systems have also been integrated with industrial manipulators via TCP/IP [4]. Recent industrial-robot research extends this trend toward sensor-rich and AI-assisted workcells; for example, stereo 3D vision can expand environmental awareness while preserving controller-level positioning accuracy [5], whereas collaborative robot measurement systems demonstrate the benefits of repeatable sensor positioning for automated inspection [6]. These approaches increase flexibility and measurement coverage, but they also introduce calibration, integration, and workcell-specific validation requirements. These studies demonstrate the value of well-defined command and communication layers, but they do not directly address transient label errors in contactless gesture streams. Environmental studies in robotic bin picking further show that illumination variations, surface optical properties, occlusions, and part geometry can degrade the performance of AI-based perception systems [7].

**Figure 1:**
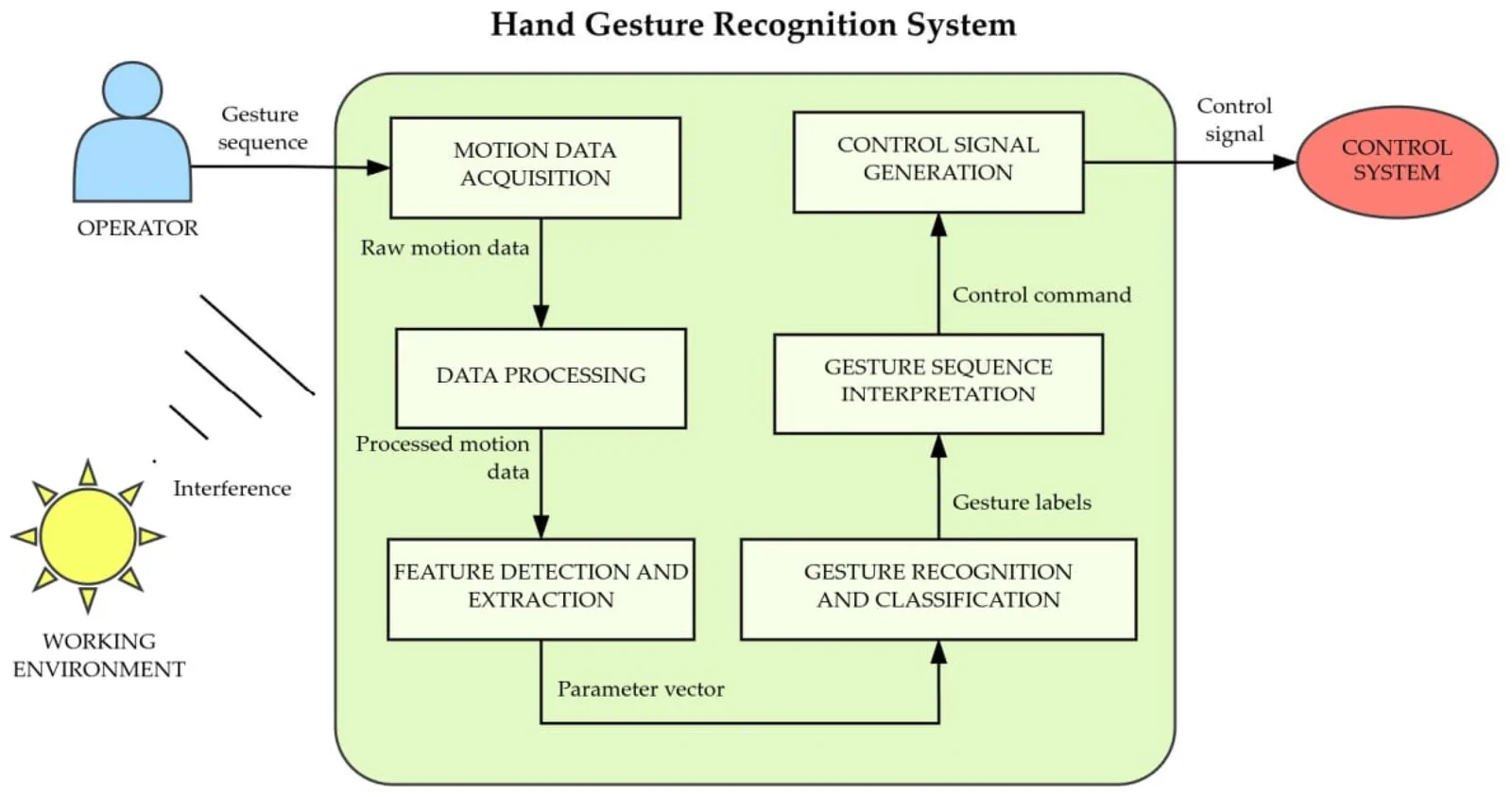
Flowchart of the processing of gesture sequences by the HGR control system.

Vision-based hand gesture recognition (HGR) for human–robot interaction (HRI) typically comprises image acquisition, hand detection or segmentation, feature extraction, and gesture classification [8,9]. Figure 1 illustrates this conventional processing chain. For industrial control, an additional validation layer is needed between classification and command execution because a transient perception error may otherwise activate an unintended robot function.

Wearable gloves and capacitive sensors can provide stable gesture-related signals [10,11], while surface electromyography supports gesture recognition in assistive and prosthetic systems [12,13,14,15]. However, these approaches require equipment attached to the operator, which may be undesirable in industrial environments. Therefore, the present study adopts contactless vision-based sensing, accepting the associated sensitivity to ambient lighting, background complexity, camera distance, and partial hand occlusion.

RGB-D and skeletal HGR studies have shown that geometric hand representations can support real-time recognition under realistic operating conditions [16,17,18]. MediaPipe extracts 21 spatial landmarks from each detected hand and has been used in real-time gesture interfaces, mobile robot control, and user-configurable HGR systems [19,20,21]. Landmark-based processing is particularly well suited to edge computing platforms, because it replaces high-dimensional full-image classification with a compact geometric feature vector [22,23].

Both classical machine learning methods and deep sequential architectures have been applied to HGR. Comparative studies have reported effective performance for methods such as KNN, LDA, and SVM [19], whereas RGB-D and dynamic gesture systems are increasingly using 3D-CNN, CNN–LSTM, ConvLSTM, or Transformer models [24,25,26,27,28,29]. Recent examples include dynamic hand-gesture teleoperation that combines depth-based MediaPipe landmarks with semantic and pose estimation for a quadruped robot equipped with a manipulator [27], and a MediaPipe–Transformer architecture for temporal gesture modeling [28]. These methods broaden the command vocabulary and capture temporal context, but their greater model complexity, sequence dependence, and computational cost can complicate deterministic deployment. Contactless control has also been extended beyond hand gestures; for example, in [30], a lightweight GhostNet–BiLSTM–attention architecture mapped facial gestures to robot commands in a Unity simulation. Facial gestures can support assistive and hands-free interaction, although performance remains dependent on facial visibility, lighting, and user-specific expression patterns. Lightweight convolutional and ensemble models have also been investigated for real-time deployment [22,31]. Although these studies establish that gesture classes can be recognized accurately, most focus primarily on recognition performance rather than on converting fluctuating label streams into deterministic industrial commands.

Gesture interfaces have been validated with mobile, assistive, collaborative, and industrial robots [1,23,32,33,34]. Gesture control has also been applied to a leech-inspired soft crawling robot using static and dynamic recognition methods [35]. This confirms the portability of natural interfaces across robot morphologies while also showing that gesture vocabularies, command mappings, and motion constraints must be validated for each target platform. Related research also emphasizes the importance of operator safety, ergonomics, and perceived predictability in HRI [36,37]. Vision-based systems remain vulnerable to non-uniform backgrounds, illumination changes, and occlusions [8,38,39], and high-performing detectors or deep classifiers do not inherently suppress short-lived misclassifications during physical transitions between gestures [40,41,42]. Wider deployment of robotic interfaces additionally requires attention to work organization, ergonomics, ethics, and legal constraints [43,44,45,46,47].

The literature therefore reveals a specific gap between gesture recognition and industrial command generation. Existing work provides mature HGR pipelines and intuitive robot interfaces, but comparatively little attention has been paid to the intermediate layer that transforms a fluctuating stream of gesture labels into a temporally validated and context-dependent control command. This gap is particularly important in industrial robotics because even a brief label fluctuation can trigger an unintended program or motion when labels are mapped directly to execution functions.

The novelty of this study lies in treating HGR output as an unverified signal that must undergo deterministic validation before entering the robot execution layer. The proposed framework integrates lightweight landmark classification with a cellular-automaton-based transition stabilizer and a two-hand command grammar. The stabilizer suppresses short-lived label fluctuations, while the grammar separates command context selection from action execution.

The main contributions of this work are as follows:

(1) A lightweight 63-feature multilayer perceptron classifier that operates on geometrically normalized MediaPipe hand landmarks;
(2) A population-based distance-filtering procedure for removing inconsistent landmark configurations from the training set;
(3) A cellular-automaton-based stabilizer that converts fluctuating classifier outputs into validated gesture transitions;
(4) A two-hand command grammar that separates command context selection from action execution;
(5) A comparative evaluation of raw classifier output, majority voting, three-consecutive-label confirmation, and the proposed CA-based stabilizer across distinct interface actions;
(6) Experimental validation using both RobotStudio and a physical ABB IRB 120 industrial robot.

Accordingly, this study evaluated the complete perception-to-control chain rather than gesture classification in isolation, with particular emphasis on temporal stability, contextual interpretation, and practical integration with an industrial robot controller.

## 2. Materials and Methods

### 2.1. Test Setup and System Architecture

**Figure 2:**
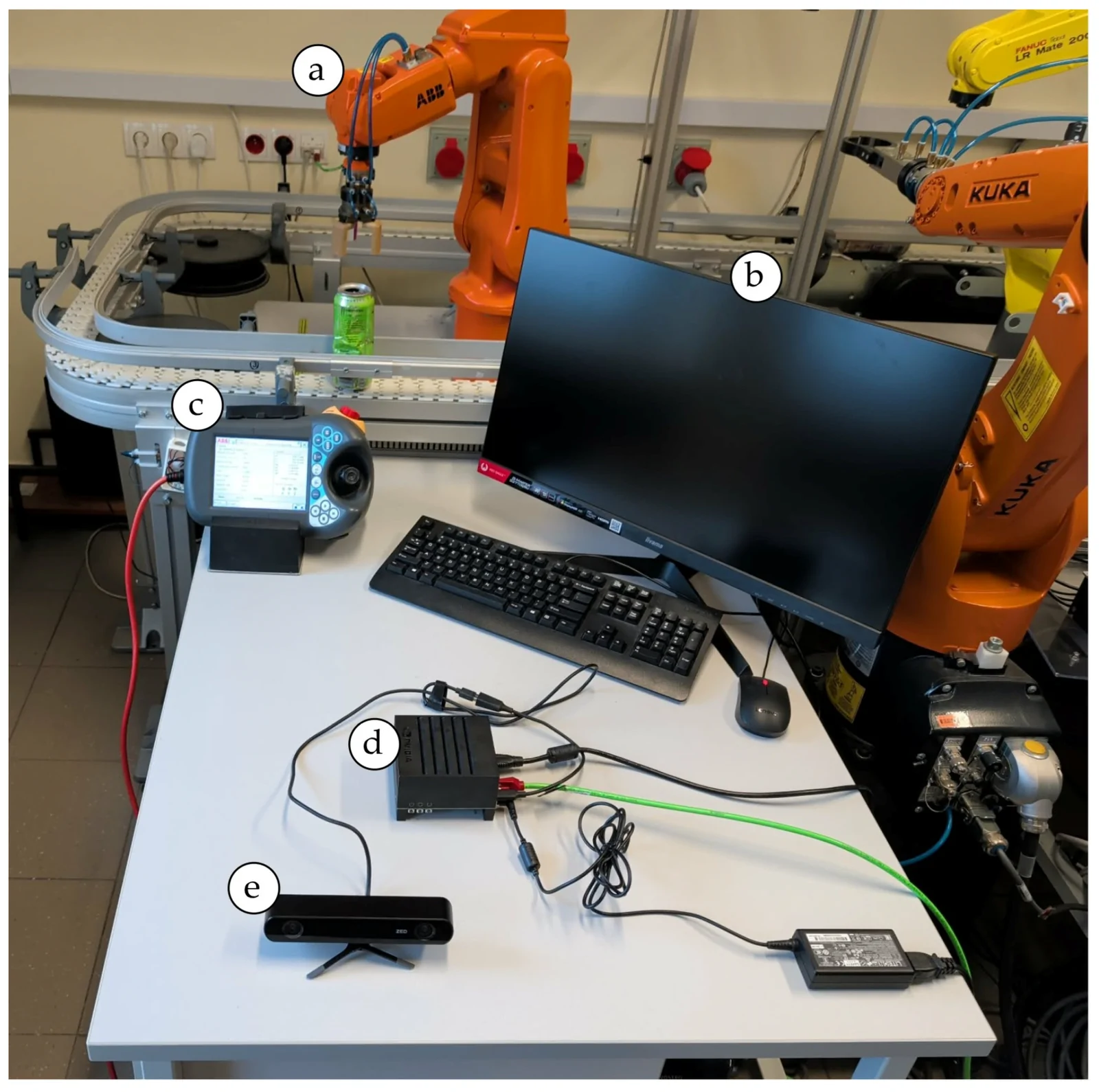
View of the experimental setup: (**a**) IRB 120 robot with a controller; (**b**) computer with a monitor for operating the GUI; (**c**) FlexPendant; (**d**) Jetson AGX Xavier; and (**e**) ZED 2 camera.

The experimental platform comprised an ABB IRB 120 industrial robot (Asea Brown Boveri, Västerås, Sweden) with an IRC5 Compact controller (ABB Robotics, Västerås, Sweden), ZED 2 stereo camera (Stereolabs SAS, Montrouge, France), Jetson AGX Xavier computer (NVIDIA Corporation, Santa Clara, CA, USA), and graphical user interface (Figure 2).

The ZED 2 camera provides RGB frames for the perception layer. A Python 3.10 application running on the Jetson AGX Xavier processes the image stream, extracts hand landmarks using the MP (MediaPipe) model, classifies gestures using an MLP, and generates command tags. The robot is controlled by a separate integration layer that uses a proxy server with the integrated ABB SDK.

The application employed a multithreaded architecture. The camera thread acquires image frames, the MP recognition thread identifies landmarks and performs normalization, the central thread performs classification and command mapping, the communication thread maintains the connection to the server, and the GUI thread displays the image, landmarks, the classifier’s result, the command queue, and the robot’s status. This separation prevents blocking among data acquisition, inference, command interpretation, and visualization processes.

### 2.2. Gesture Dictionary and Hand Roles

**Figure 3:**
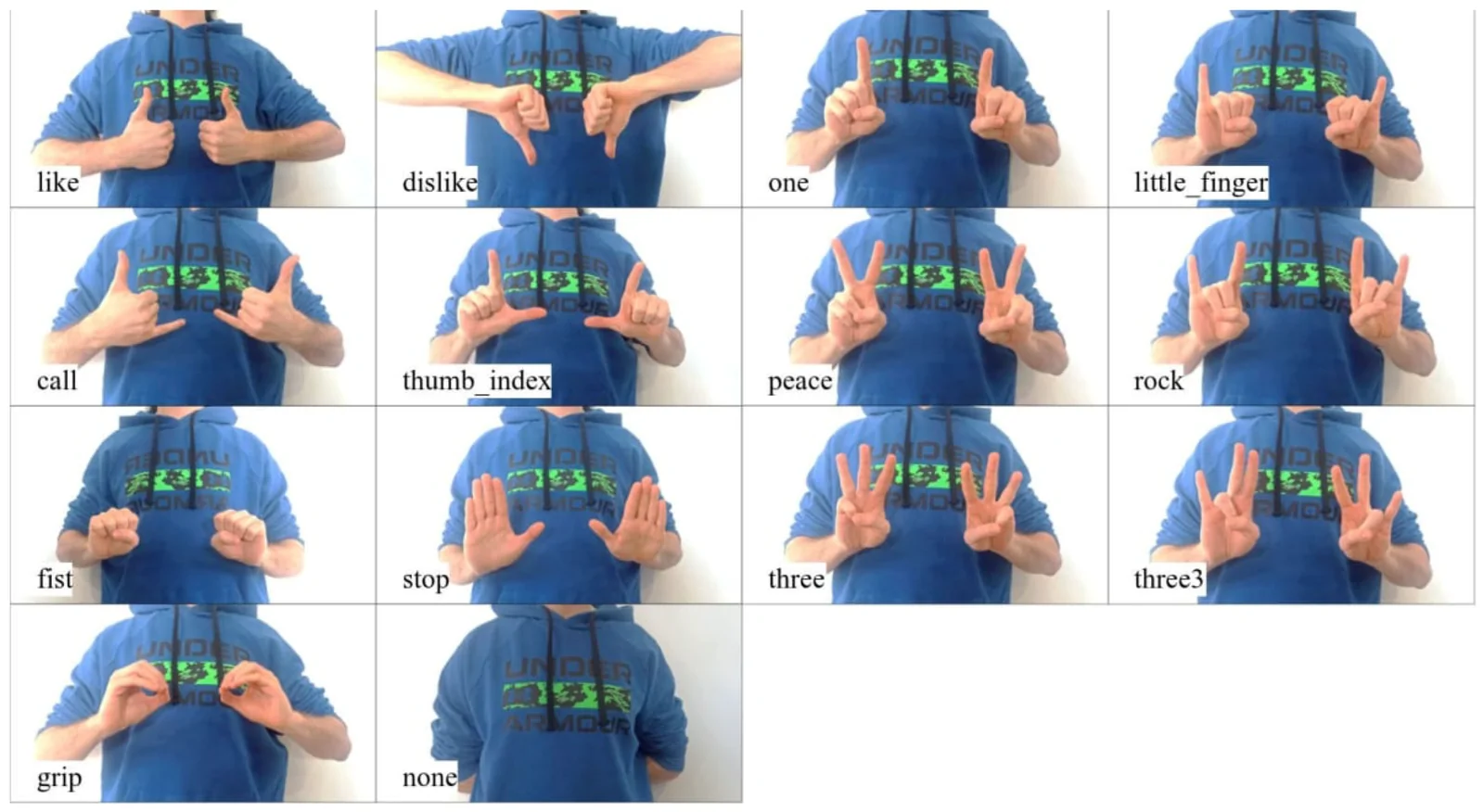
Gestures set.

The gesture dictionary comprises the following 13 active labels: “like”, “dislike”, “one”, “little_finger”, “call”, “thumb_index”, “peace”, “rock”, “fist”, “stop”, “three”, “three3”, and “grip”. The additional operational label “None” denotes the absence of a valid gesture, which is assigned when a hand cannot be detected in the frame, or may be assigned during the stabilization of the stream of classified labels over time (Figure 3). This set was selected on the basis of visual distinctiveness, motor feasibility, and suitability for constructing short sequences of commands. Gestures are not treated as individual commands, but as transitions in a two-handed control language.

The interaction model distinguishes between the main hand and the command hand. The main hand triggers the query—that is, the command context—while the command hand performs actions within that context. The same transition can represent GUI navigation in one query and a robot’s movement in another. This approach increases the number of available commands without increasing the number of static gestures. When a context is activated or a command is entered, the system does not impose any restrictions on the maximum duration of the gesture. However, it is necessary to keep the gesture within the camera’s field of view for the entire duration of the context or while the command is being executed.

### 2.3. Multithreaded System Architecture

**Figure 4:**
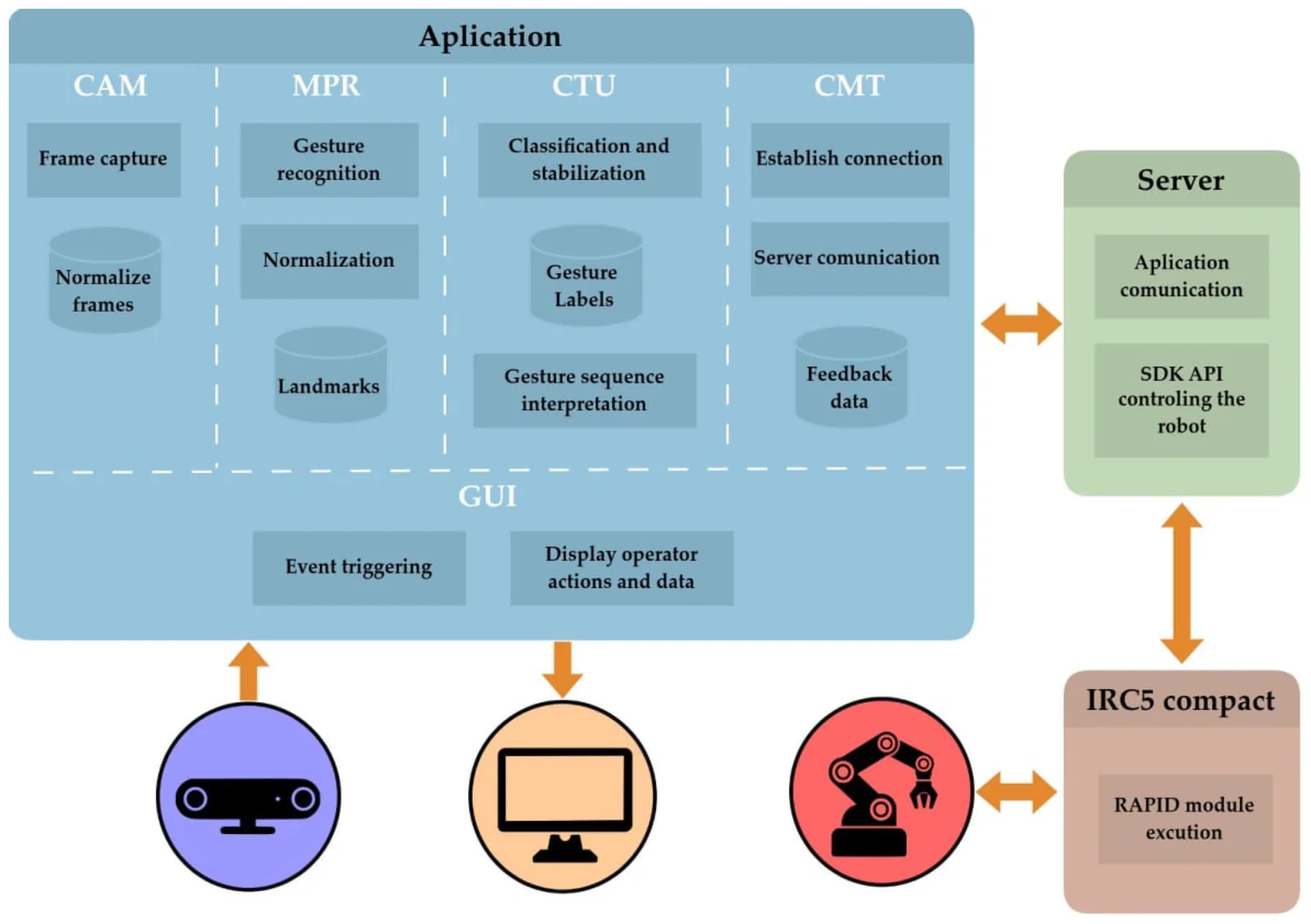
Architecture of the HGR control system.

Multithreading was required to separate the frame reading process; hand landmark detection using the pretrained MediaPipe model; the label classification process; the control, management, and external communication systems; as well as the graphical user interface (Figure 4). Multithreading substantially improved platform performance, but it also introduced the problem of asynchronous data transfer between threads and increased the complexity of the final implementation.

As shown in Figure 4, the system comprises three layers.

- An application that is divided into five threads: CAM, MPR, CTU, CMT, and GUI;
- A server that manages the robot’s communication and software operations;
- Control programs in the IRC5 Compact controller.

The camera operates at 30 Hz with a resolution of 1280 × 720 pixels. Combining frame acquisition with the detection process performed by the MP model caused delays. Therefore, these processes were separated into two independent threads: the Camera (CAM) thread and the MediaPipe Recognizer (MPR) thread.

The Communication Thread (CMT) module was also separated from the Central Unit Thread (CTU). This separation prevents the communication process, and consequently the entire application, from being blocked. The CTU is responsible for classifying gestures using an MLP model, stabilizing the label stream using a cellular automaton, and interpreting successive gesture sequences and mapping them to control function tags.

The GUI thread handles events generated by the operator and displays the video stream along with synchronized landmarks, classification results, and the results of their stabilization. The GUI is also responsible for displaying control prompts, the status of the query being built to trigger the control function, and feedback received from the robot and the server. The server interprets commands and their arguments and passes them to the appropriate functions for controlling the robot.

### 2.4. Application Pipeline System

**Figure 5:**
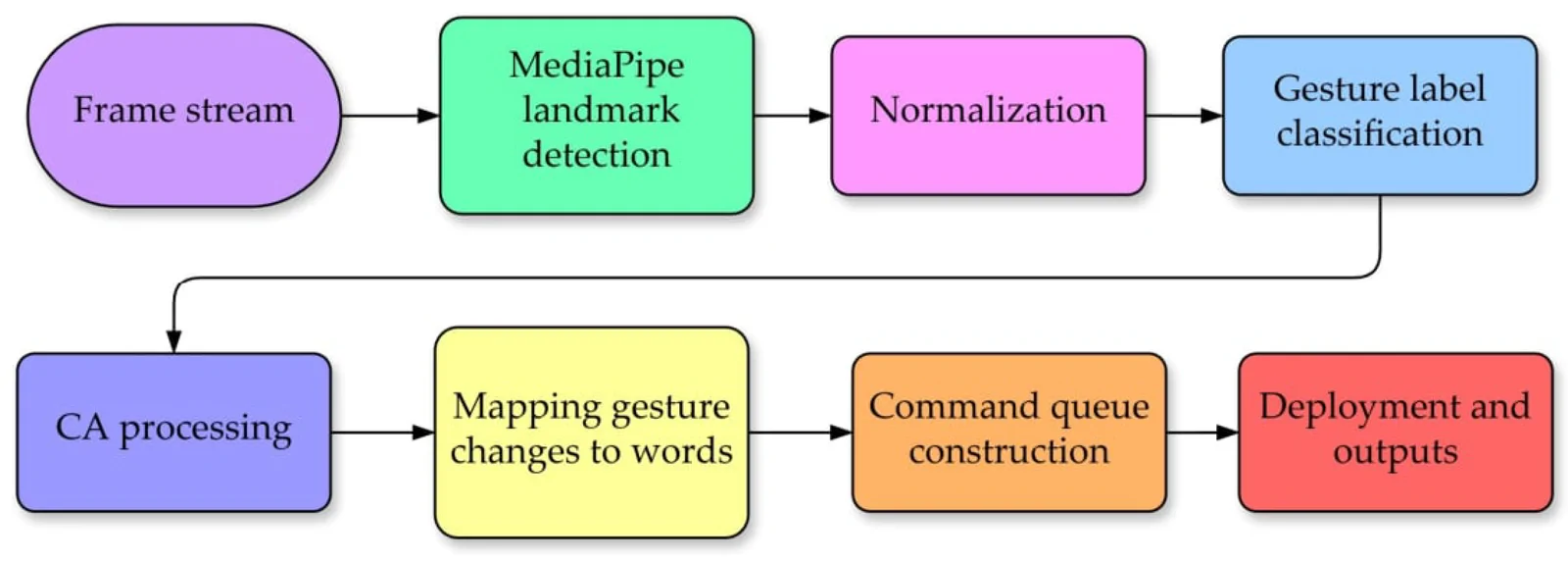
HGR system pipeline stages.

A stream of video frames is acquired at the application input. For each successive frame, the MP model detects hand landmarks, which are then subjected to a four-step normalization process consistent with the procedure used during the preparation of the training data. The normalized input landmarks are fed into an MLP model developed specifically for the system, which classifies the gesture labels. Classification generates a discrete stream of class labels, which is subsequently stabilized using a cellular automaton (CA). The system analyzes successive changes in gesture states and, based on them, selects the corresponding words or control function tags. A syntactically valid logical sequence corresponding to a defined command generates an output instruction. Depending on the type of command, it is sent to the robot’s control server or used to control the graphical user interface (Figure 5).

### 2.5. Data Preparation and Landmark Normalization

**Figure 6:**
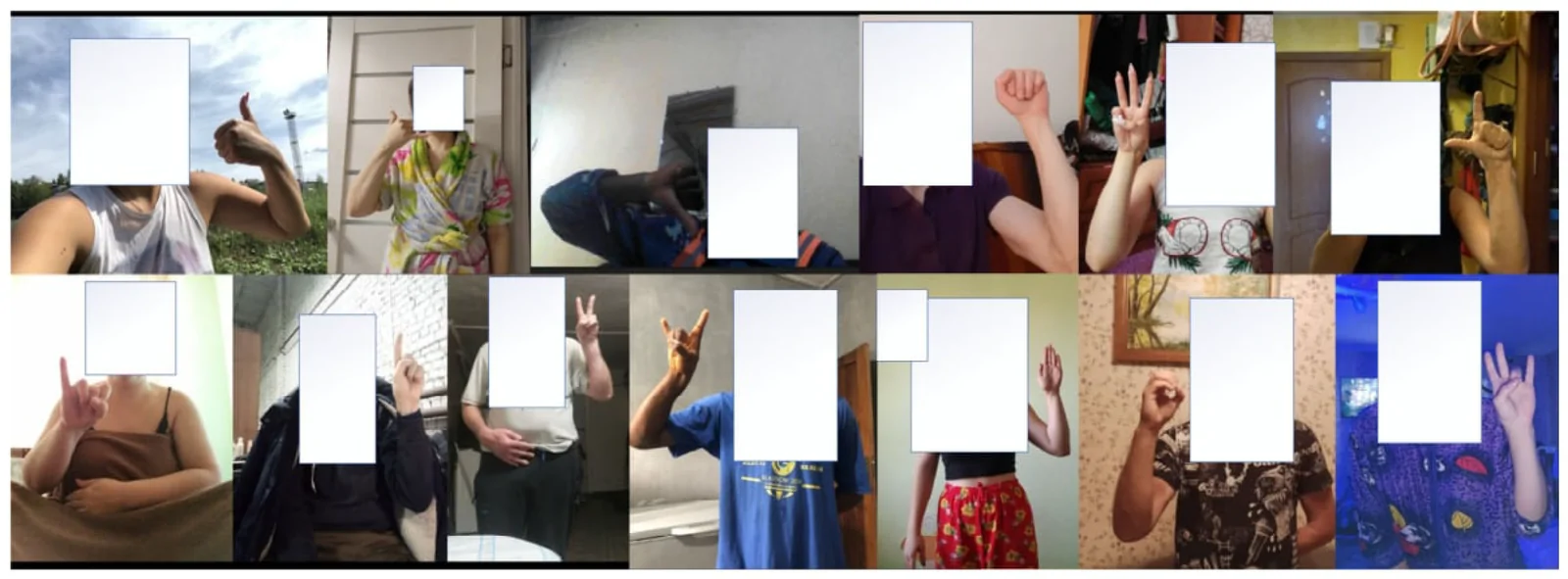
Thirteen sample gestures from the HaGRIDv2 dataset that was used in the training of the classifier [48].

The training data were generated from the HaGRIDv2 dataset (Figure 6). A reduced subset of 512p images was used because the full dataset is substantially larger than required for this feasibility study. For each class, 2000 images were randomly sampled. Each image was processed using MediaPipe Hand Landmarker to generate a vector of 21 landmarks, each represented by x-, y-, and z-coordinates. The classifier’s input is therefore a 63-dimensional vector, not the full image.

Raw landmarks depend on translation, scale, distance from the camera, and hand orientation, thereby producing an inconsistent dataset. To improve consistency, a four-step normalization process was applied to each landmark individually. All points are centered relative to the wrist. Next, the point cloud is scaled by the distance between the wrist and the metacarpophalangeal (MCP) joint of the middle finger. Samples from the left hand are mapped to the right hand. Finally, the point cloud is rotated relative to the wrist by an angle between the coordinate system axis and the direction vector determined by the resultant coordinate of the metacarpophalangeal joints of the index finger and little finger.

### 2.6. Filtering of Inconsistent Training Samples

**Figure 7:**
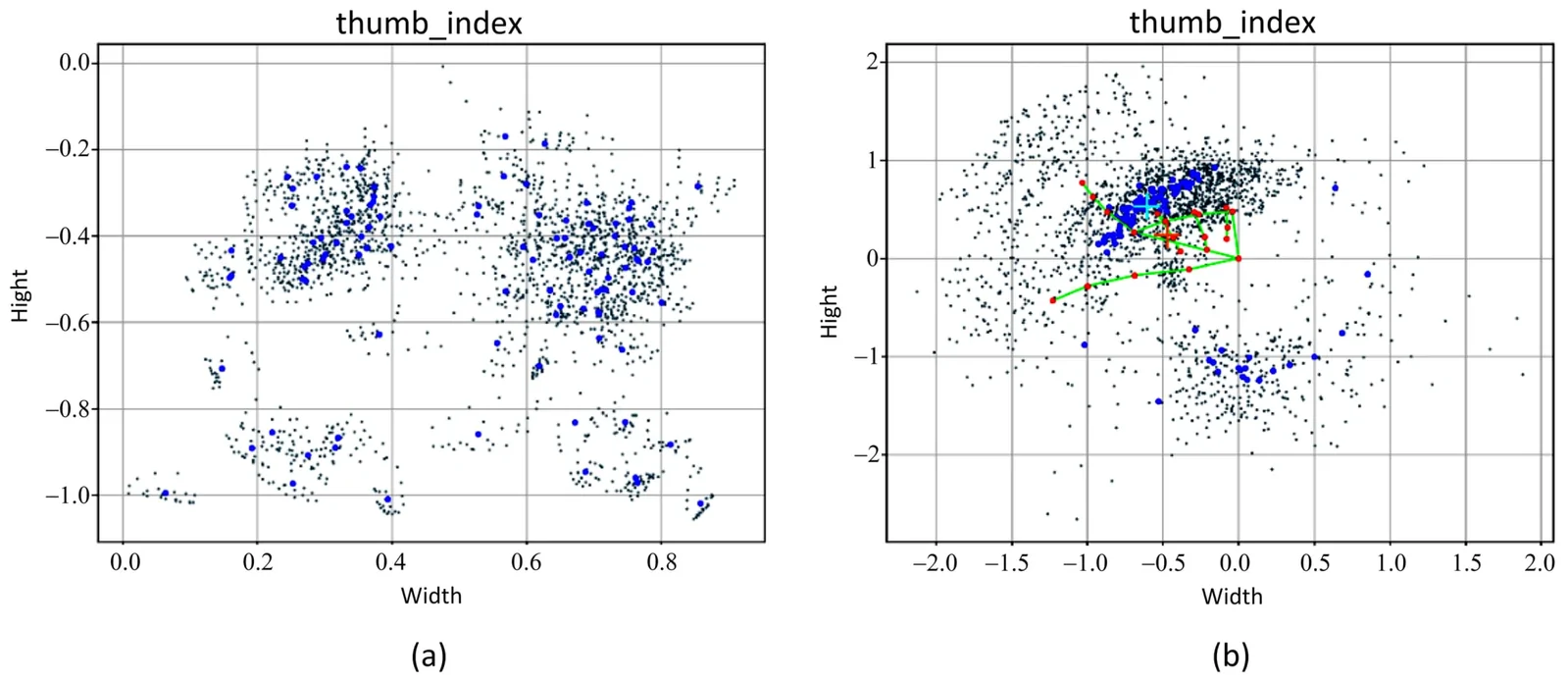
Comparison of hand landmark distributions before and after geometric normalization for 100 thumb_index gesture samples: (**a**) raw landmark distributions obtained using MediaPipe Hand Landmarker; (**b**) distributions after normalization of translation, scale, hand side, and orientation. ·—consecutive landmark points; ●—geometric center points of consecutive landmarks. A landmark representing the weighted averages of the individual landmarks was superimposed on the population. The centroid of the mean landmark configuration is marked with a red cross, while the center of the largest cluster of points identified by the DBSCAN algorithm is marked with a blue cross. The reduced dispersion in panel (**b**) illustrates the effect of the normalization procedure used.

After normalization, some samples may still contain incorrect or inconsistent landmarks, usually caused by the detection of the wrong hand, background objects, or partially visible gestures. These samples were removed using a population distance filter. For each class, the geometric centroid of the landmark cloud is compared with the center of the population of the largest cluster of that class, identified using the DBSCAN algorithm. Samples that exceed the selected threshold are rejected as outliers (Figure 7).

**Figure 8:**
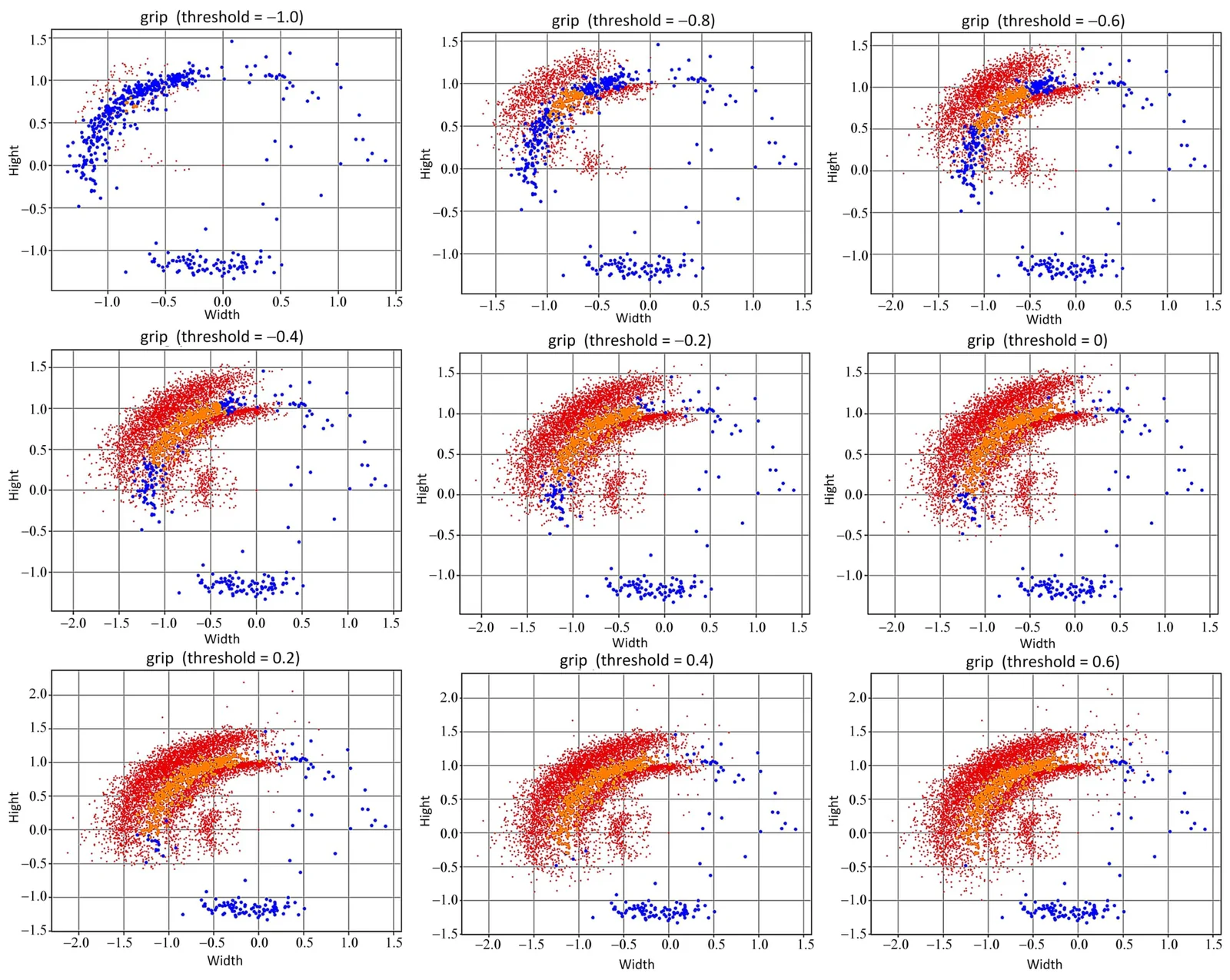
Filtration process (changing the threshold parameter within the range of –1 to 0.6); blue points—geometric centers of landmarks that did not pass through the filter; orange points—geometric centers of landmarks that passed through the filter; red points—all landmark points that passed through the filter.

**Figure 9:**
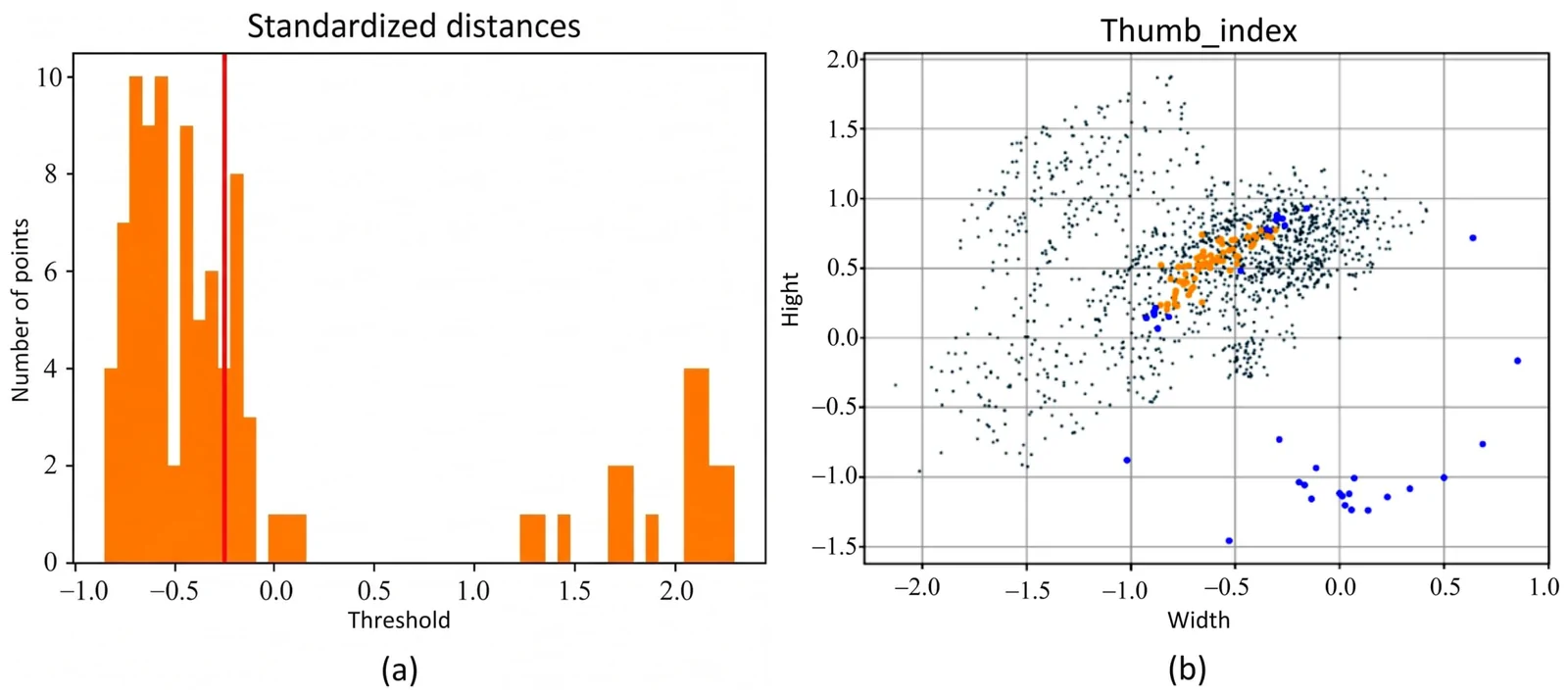
An example of the standardization process for a minimum-distance filter applied to a group of 100 landmarks from “thumb_index” gestures with a threshold parameter of −0.25. (**a**) The distribution of vectors between the geometric centers and the central geometric center; the red line indicates the value of the threshold (−0.25). (**b**) A plot of all 2100 points, with their geometric centers marked in blue and orange; the geometric centers of the samples that passed through the filter are marked in orange.

Filtering was performed by analyzing the normal distribution of vector values between the geometric centers of individual instances of the group and the geometric center of the entire population. The acceptance range for samples belonging to the standardized population was defined by the “threshold” (Figure 8). A threshold of −0.25 was selected as a compromise between defect removal and preserving the number of training examples (Figure 9). This step reduced the size of the set but improved the consistency of the class population. The changes in sample size are summarized in Table 1. Because some HaGRIDv2 images contained more than one detectable hand, landmark extraction produced 29,347 hand instances from 26,000 source images. The extracted set therefore corresponded to 112.9% of the original image count, representing an increase of 12.9%.

**Figure 10:**
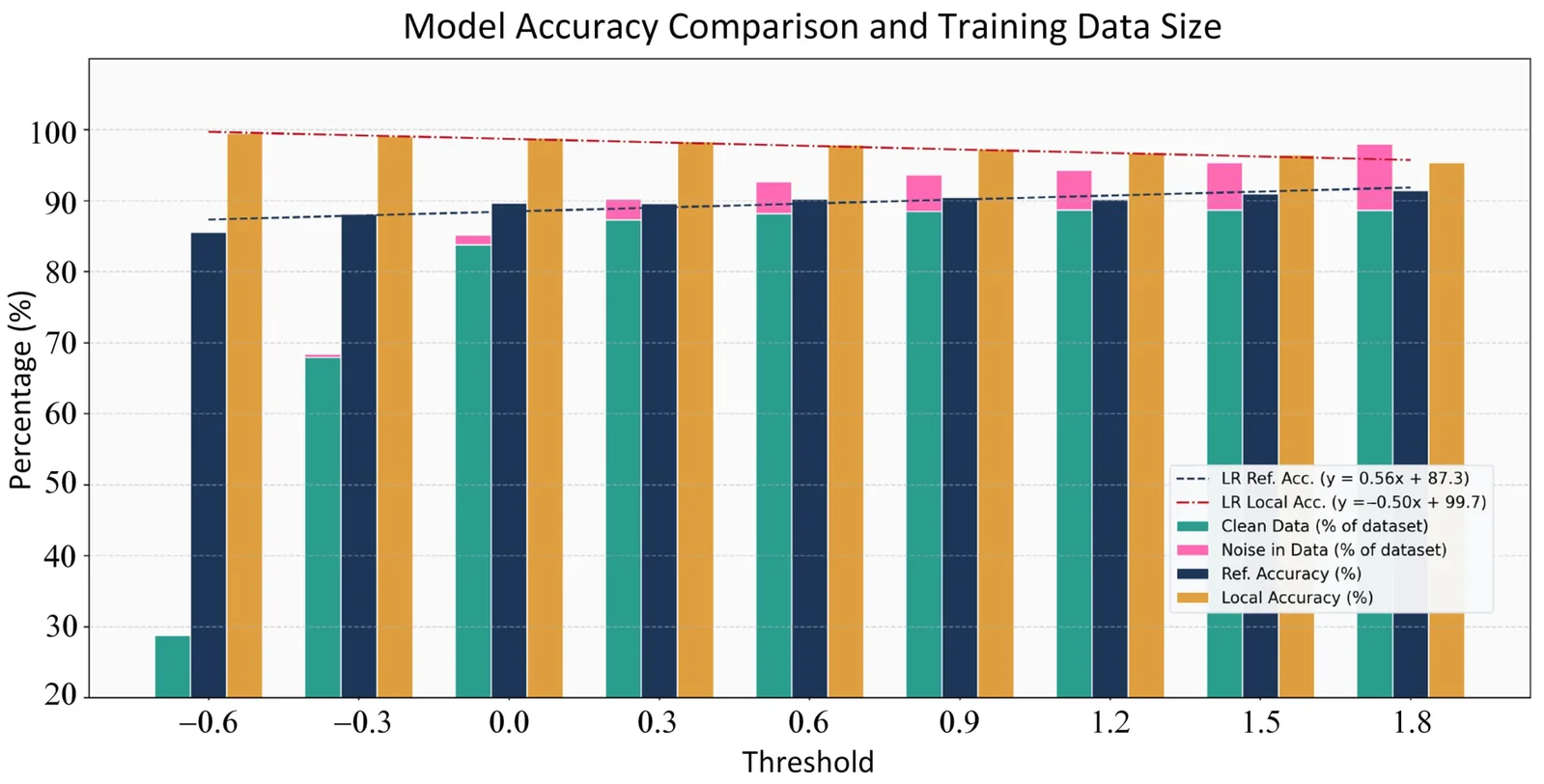
The impact of the filtering threshold on the retention of training data, the mismatch level, reference accuracy (comparing trained models on a reference set consisting of 500 samples per class), and local accuracy.

The filtering threshold was selected using two complementary evaluation perspectives. A threshold of −0.25 was selected for the final model because it favored unambiguous and repeatable gesture configurations while maintaining sufficient training samples for all classes. This setting did not maximize nominal reference accuracy; however, the differences in accuracy across a broad range of thresholds were relatively small. This choice was particularly relevant to the present study because reducing label noise helped distinguish errors originating from gesture classification from transient disturbances handled by the downstream temporal stabilization stage. Accordingly, −0.25 should be interpreted as an empirically selected operating point for the present dataset and experimental objective rather than as a universally optimal threshold (Figure 10).

The dataset obtained after filtering was used to train the MLP model. The model receives a 63-dimensional feature vector comprising the *x*-, *y*-, and *z*-coordinates of 21 hand landmarks. The model architecture comprises three fully connected layers containing 128, 256, and 128 neurons, respectively. The hidden layers use the ReLU activation function, batch normalization, and dropout with coefficients of 0.4, 0.3, and 0.3. The output layer contains 13 neurons with a softmax function, each corresponding to a specific class. For model training, 70% of the data was used as training data, 15% as validation data, and 15% as test data. The model was trained using the Adam optimizer and the categorical cross-entropy loss function. Training was conducted for up to 100 epochs with a batch size of 64. The Early Stopping and ReduceLROnPlateau mechanisms were used to limit overfitting and adjust the learning rate based on the validation error.

**Figure 11:**
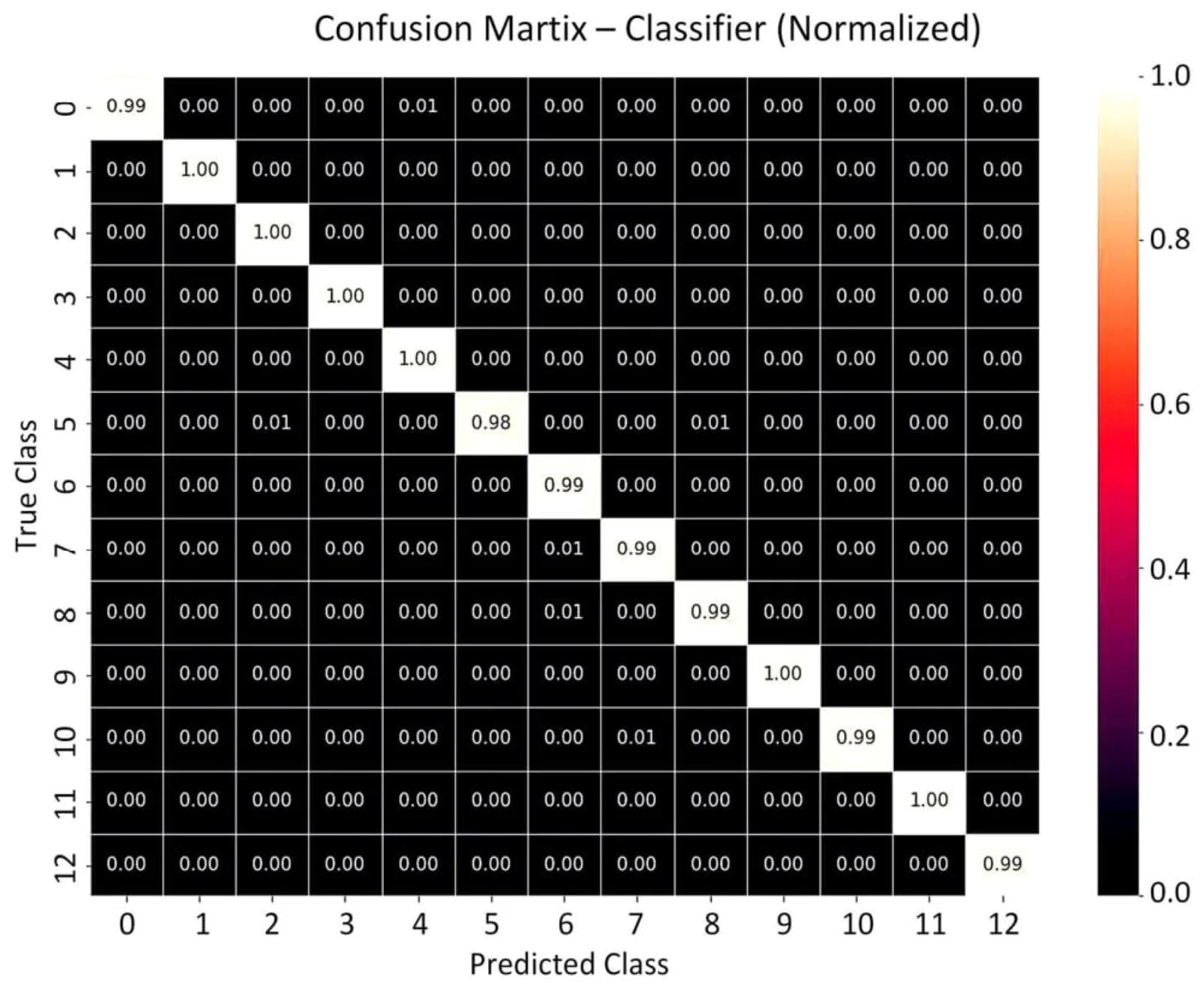
Confusion matrix obtained using 15% of the test dataset and generated using the trained MLP model.

Figure 11 shows the row-normalized confusion matrix obtained for the held-out test subset using the trained MLP classifier. The matrix is strongly diagonal, indicating that the selected gesture classes are highly separable. The average value along the main diagonal is approximately 99.46%, which corresponds to the mean recall per class. Misclassifications only occurred sporadically and were limited to a small number of visually similar gesture classes. The results confirm that the proposed 63-dimensional landmark representation preserves sufficient geometric information to accurately distinguish the 13 selected hand gestures. Accordingly, most samples were assigned to the correct classes, with few misclassifications across classes. The resulting distribution supports the effectiveness of the developed model in distinguishing among the analyzed classes. The classification metrics reconstructed from the row-normalized confusion matrix are summarized in Table 2.

As precision, macro-F1 score, and overall accuracy were not retained in the exported results, classification performance is reported only using metrics directly derivable from the normalized confusion matrix.

### 2.7. Application and Control System

Partitioning the application into a multithreaded architecture isolated computationally intensive operations, ensuring smooth GUI operation and stable control based on a stream of classified labels. However, the use of multithreading required the system to be expanded to include a timestamp mechanism and temporary data buffers to store data being transferred between threads. The key challenge in terms of synchronization was handling the GUI input data and, in particular, the smooth visualization of the camera image with the landmark grid superimposed on it.

The classified-label stream was more challenging for control, as it contained a number of disruptions that adversely affected the system’s operational safety. The CA model was used to stabilize the transitions between labels and eliminate interference in the data stream.

#### 2.7.1. Cellular-Automaton-Based Transition Stabilizer

**Figure 12:**
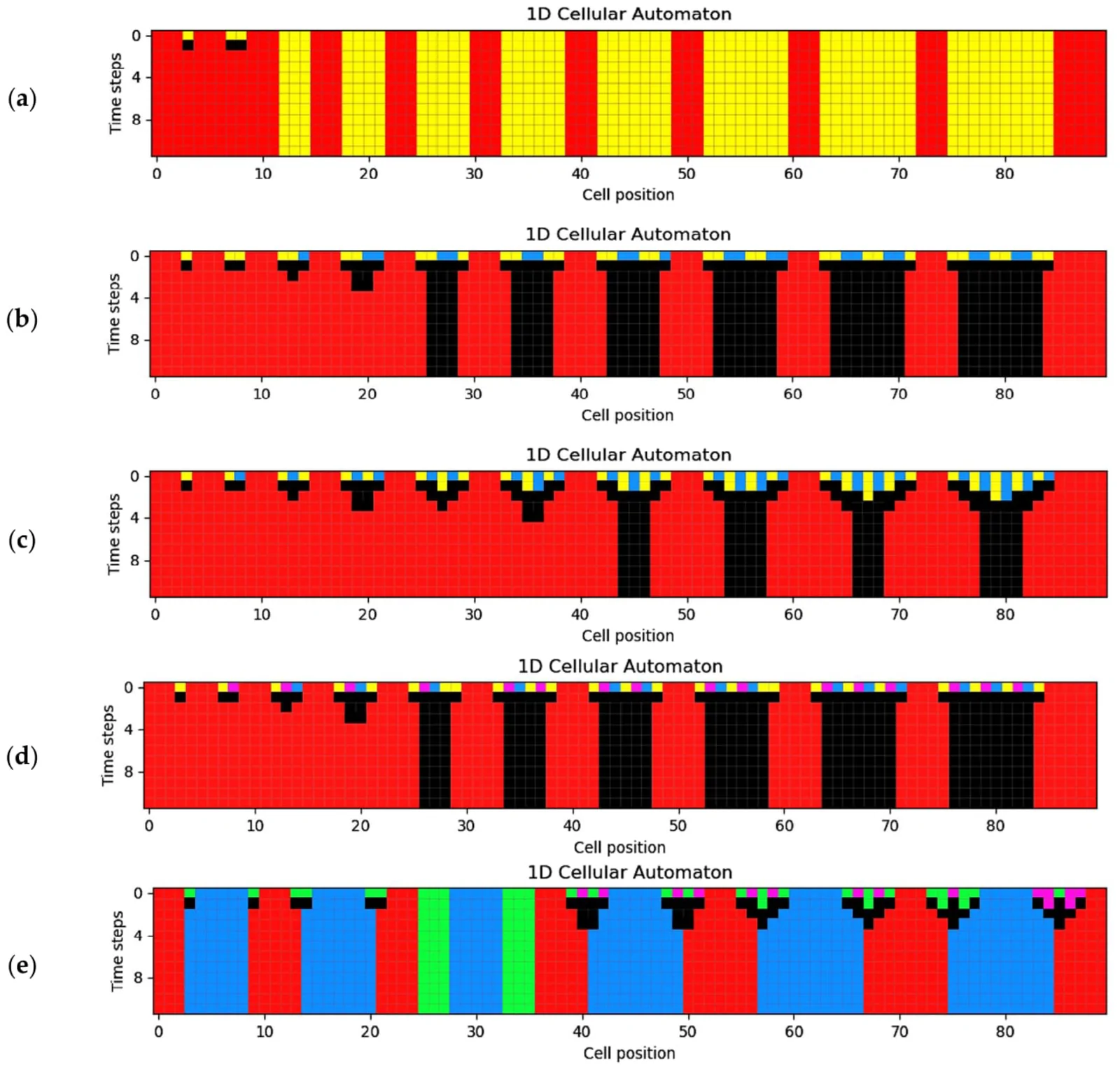
Sample waveforms of sample gestures at successive time steps: (**a**) gesture maintained for longer than 300 ms; (**b**) alternating between two gesture labels every 200 ms (yellow and blue); (**c**) alternating between two gesture labels every 100 ms (yellow and blue); (**d**) alternating among three gesture labels every 100 ms (yellow, blue, and purple); (**e**) transition between two stable gesture labels affected by intermediate disturbances. Red and blue indicate the stable gesture labels, whereas green and purple represent disturbances occurring during the transition (e.g., a too-slow transition from red to blue). Black—no state or stable state the “None” label.

The model was defined by the following set of rules for a one-dimensional cellular automaton processing a block of 5 cells, the central cell of which receives a new state (Figure 12):

(1) State Preservation: If there are at least 3 identical cells within a range of 2 cells, including the cell being analyzed, the cell retains its state.
(2) Death: If a cell cannot be sustained, it dies, leaving an empty space behind.
(3) Interruption: If there are 4 cells within a range of 2 cells—including the cell being processed—that are either empty or classified as “none,” a cell corresponding to the “none” label is created.
(4) Spread: If a cell is empty and there are two identical cells on one side of it, the cell takes on their state.
(5) Boundary: Cells that extend beyond the simulation area assume the state of the current cell.

Rule (1) specifies the minimum number of samples required to maintain the status. All constant strings with a length of 3 or greater will be preserved and will not be modified. Rules (2) and (4) affect the stabilization of disturbances and uneven transitions in the classification. Rule (3) forces the system to generate interruptions in areas of interference that are too large, which the system interprets as the hand having left its field of view. Rule (5) ensures that the values of the boundary cells are preserved, so that incoming new cells are not blurred relative to the active gesture and do not block the label.

The sample buffer stores the preceding 3 s of the system state, which, for a sampling time of 100 ms, results in a total of 30 samples. The CA model is responsible for processing the 7 most recent samples at each sampling interval (100 ms). During functional testing, the model suppressed short-term label changes in accordance with the defined rules, introducing a validation delay of approximately 300 ms.

#### 2.7.2. Gesture Words and Command Sequences

Transitions between gestures in a stream of class labels are called words. The application distinguishes between 15 words, consisting of 13 classifier labels, the “None” label (indicating no gesture), and the so-called “unique gesture”—the asterisk—which is interpreted as any gesture. The function for activating a new word, *f_new_*, is created by selecting a function that maps the last active state, *S_last_*, and the two most recent gestures that define the word.

(1) *f*(*S_last_*, *g_i_*_−1, _*g_i_*) = *f*(*S_last_*,w) = *S_new_*,

where

*f*—mapping function;

*S_last_*—latest active status;

*S_new_*—new state;

*g_i_*, *g*_*i*−1_—the two most recent gestures;

*w*—transition between two gestures.

However, the gestures are replaced by the word

(2) *w* = *g_i_*_−1_ → * g_i_*

Successive words form sequences interpreted as a query, which is displayed in the GUI in the form of a queue of decoded words depending on the specific context. For each context, there is a limited number of words that can be decoded, which is based on the number of available gestures. A combination of 13 labels and “None,” with no repetitions, results in a total of 182 possible combinations per context. The application in question displays a tree of six contexts, including one parent “root” from which the remaining five branches—which are not correlated with one another—extend. As different contexts are combined, the number of possible combinations grows exponentially. The application uses a limited set of combinations for all contexts. Defining all combinations each time is time-consuming, which is why notation representing a unique gesture was adopted, allowing for the declaration of all undeclared combinations. When reading a word, 4 matches are checked in the following order:

(3) *g_i_*_−1_ → *g_i_*

(4) ⋆ → *g_i_*

(5) *g_i_*_−1_→ ⋆

(6) ⋆ → ⋆

**Figure 13:**
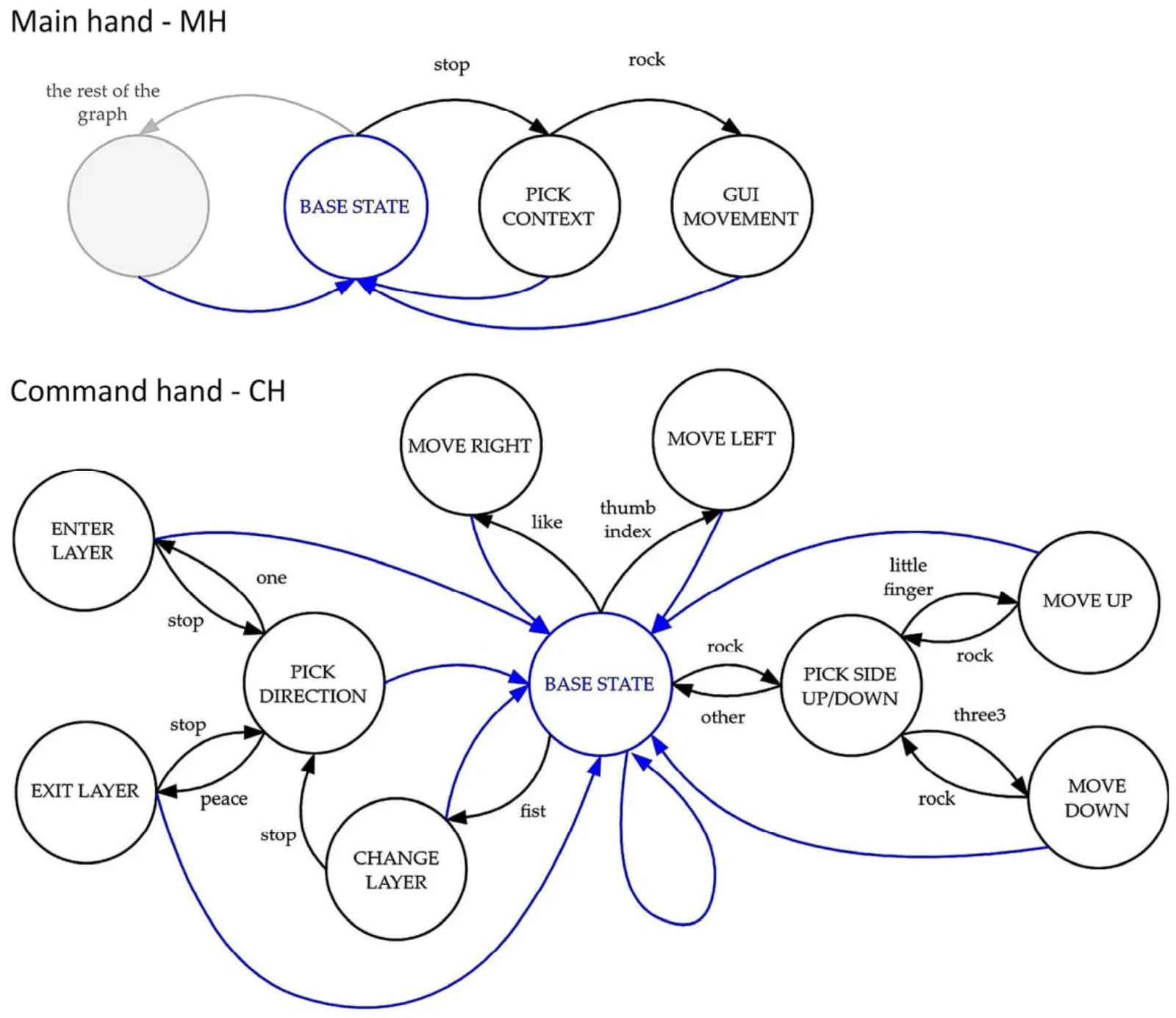
A complete transition map for the GUI motion context performed by the input (“command”) hand (The return to the baseline state is highlighted in blue. A simplified diagram of the remainder of the graph is shown in gray.).

A mapping database created in this way allows for the definition of only the vocabulary needed within the specific context in which it is used. To achieve this, three transitions must be defined: the base, entry, and exit states. In the app, navigating between contexts is limited to the primary hand, which makes the work easier and allows users to construct complete sentences more quickly and read the hints in the GUI that indicate the active context. A collective space Q is thus created, which has the structure of a graph with nesting into successive layers, and to which the operator in the final control system has access. This space is also predefined and deterministic, which contributes to the system’s stability. Branches allow users to define a query through a series of steps. Creating transitions with one or more possible outcomes allows users to select items from a template—such as choosing a program to run on an ABB robot from a list of available programs, or selecting the robot to which the server is to connect. This also allows the final instruction to be repeated multiple times without having to perform all the actions again (Figure 13).

## 3. Results

### 3.1. Verification of the Correct Operation of the Developed Software

**Figure 14:**
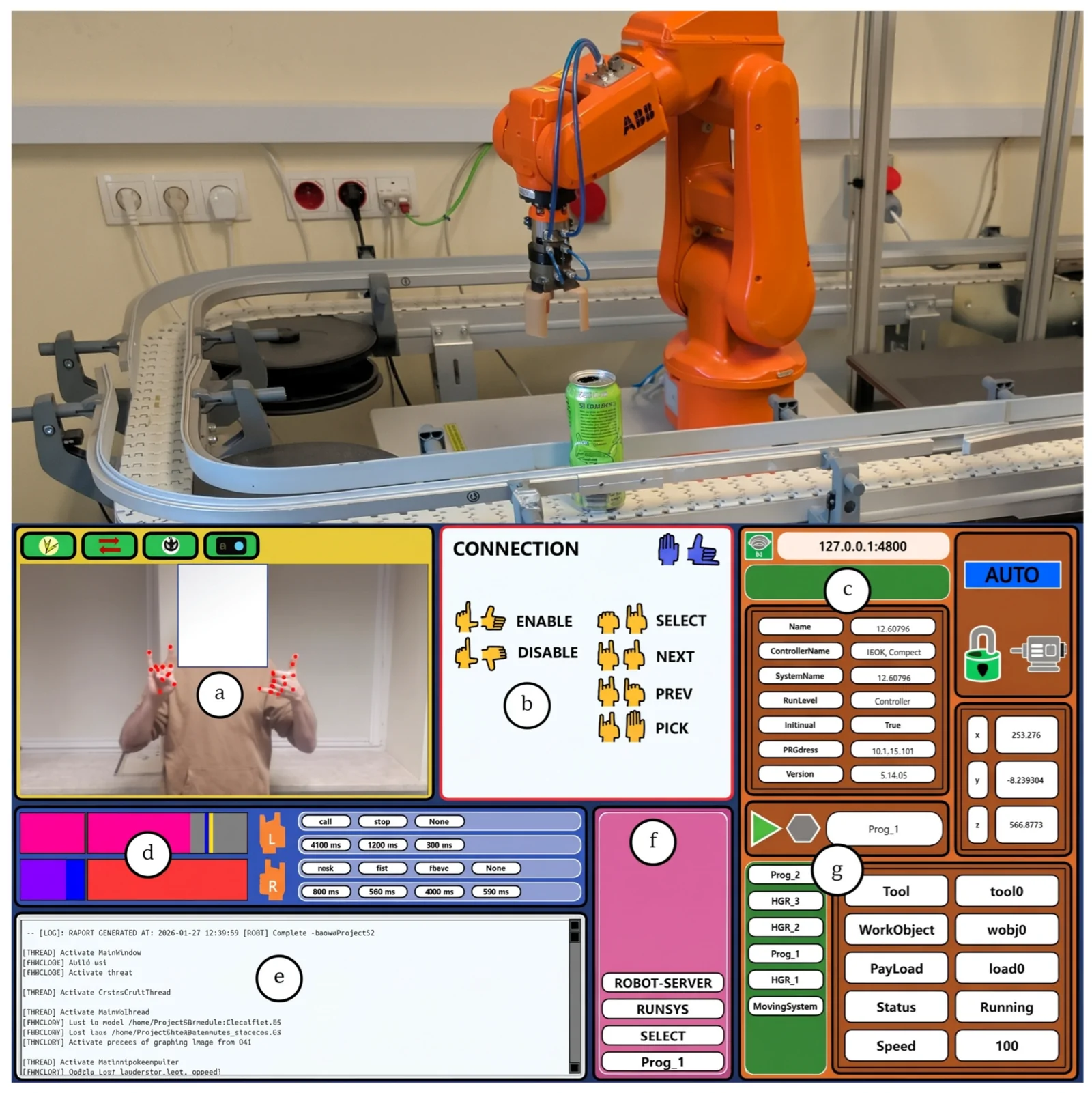
View of the test setup and GUI application: (**a**) camera image and configuration buttons; (**b**) contextual hint panel; (**c**) connection configuration panel; (**d**) discrete signal stream of classified gesture labels; (**e**) log console; (**f**) command queue; and (**g**) robot control panel.

The application was tested on a virtual workcell in RobotStudio 2025 and on a real test setup featuring an ABB IRB 120 robot (Figure 14). The experiments were designed to verify the correctness of the programs triggered by the system, the feedback retrieved by the SDK API from the robot via the server, and the direct control of the robot’s end-effector in a Cartesian coordinate system using gestures.

### 3.2. Connection Test

**Figure 15:**
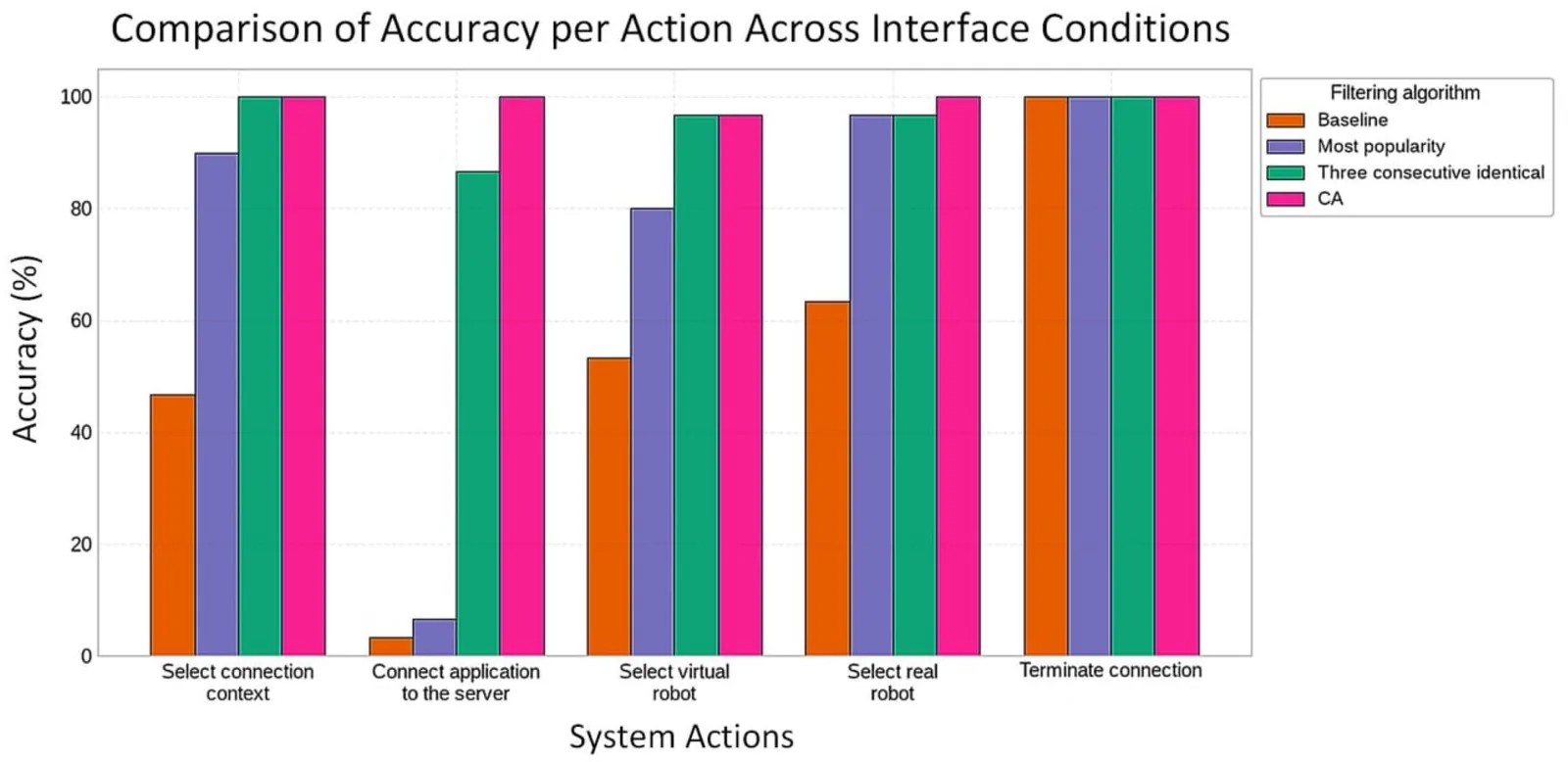
Success rates of five interface actions obtained using four temporal-processing algorithms, with 30 repetitions per action.

**Figure 16:**
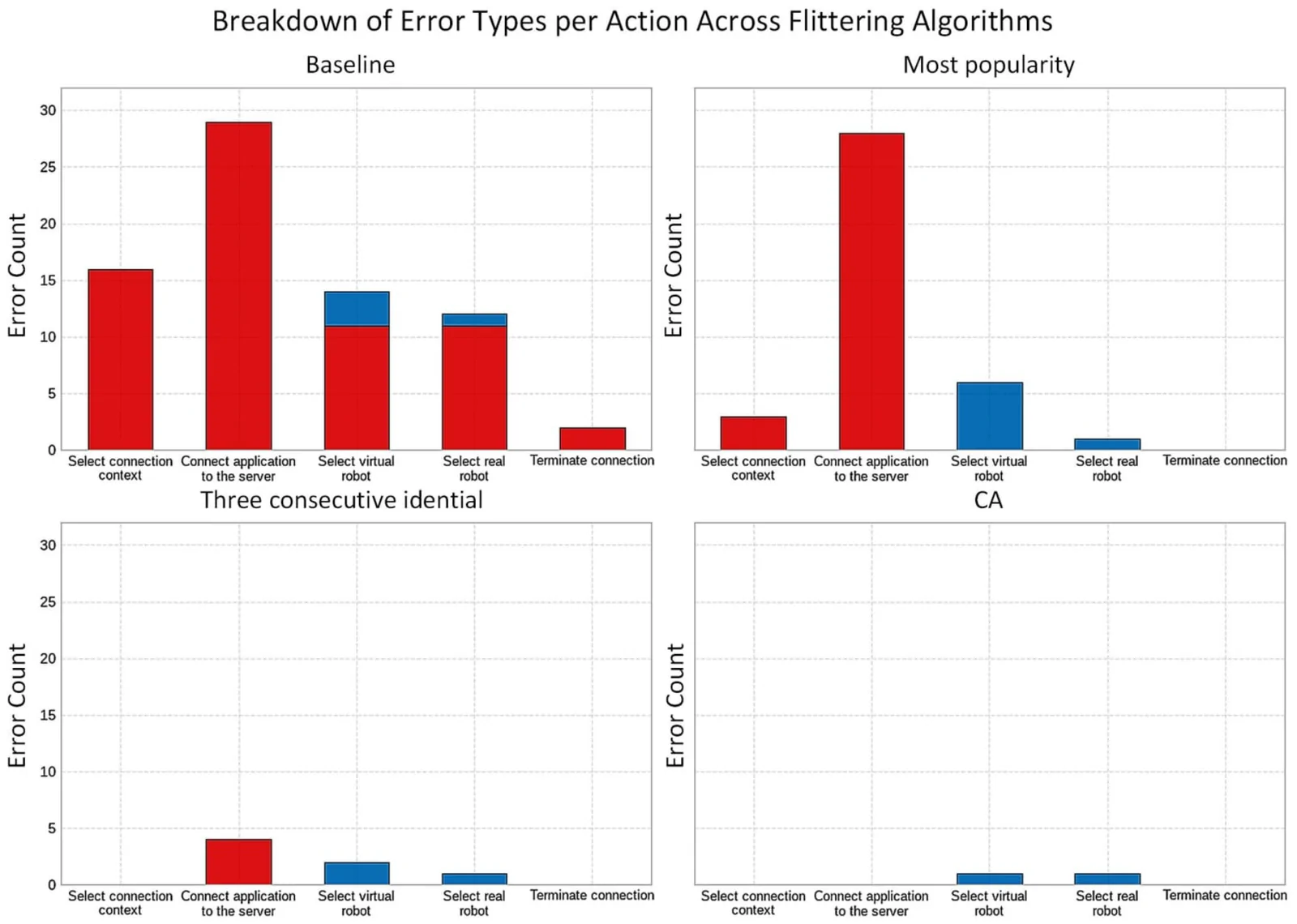
Distribution of errors across the evaluated temporal-processing algorithms. Red bars indicate system interpretation errors that prevented the requested action from being executed; blue bars indicate operator errors.

The connection test repeatedly evaluated the entire control process through five phases. Phase 1 establishes a connection between the application and the server. Phase 2 scans the network to detect available robot controllers (both real and virtual). Phase 3 selects the target controller and establishes a connection with it. Phase 4 monitors for control commands from the server. Phase 5 terminates the connection to the server and the robot controller. The success rates obtained over 30 repetitions per action are presented in Figure 15, whereas the corresponding error distributions are shown in Figure 16.

During testing, when performing the “thumb index” gesture in “Connect the application to the server”, the system recognized it as a different gesture (labeled “one”). This error was likely caused by holding the hand at different angles. During the tests, a critical angle was observed, which affected correct recognition of the gesture.

The observed system interpretation error was associated with confusion between the “thumb_index” and “one” gestures at specific hand orientations. Separately, operator errors occurred when an incorrect gesture was performed or the required sequence was not completed.

The “Baseline” variant enabled control for some highly similar gestures (e.g., “stop” → “call”, “Select connection context”); however, it did not provide sufficient stability. For gestures with more distinct characteristics (e.g., “thumb index” → “like”, “Connection application to server”), filtering based on the majority-voting method helped to reduce some of the observed errors. The three-consecutive-identical-label method achieved results comparable to those of the CA method, but was less effective for signals characterized by a higher level of noise.

### 3.3. Robot Program Control Test

**Figure 17:**
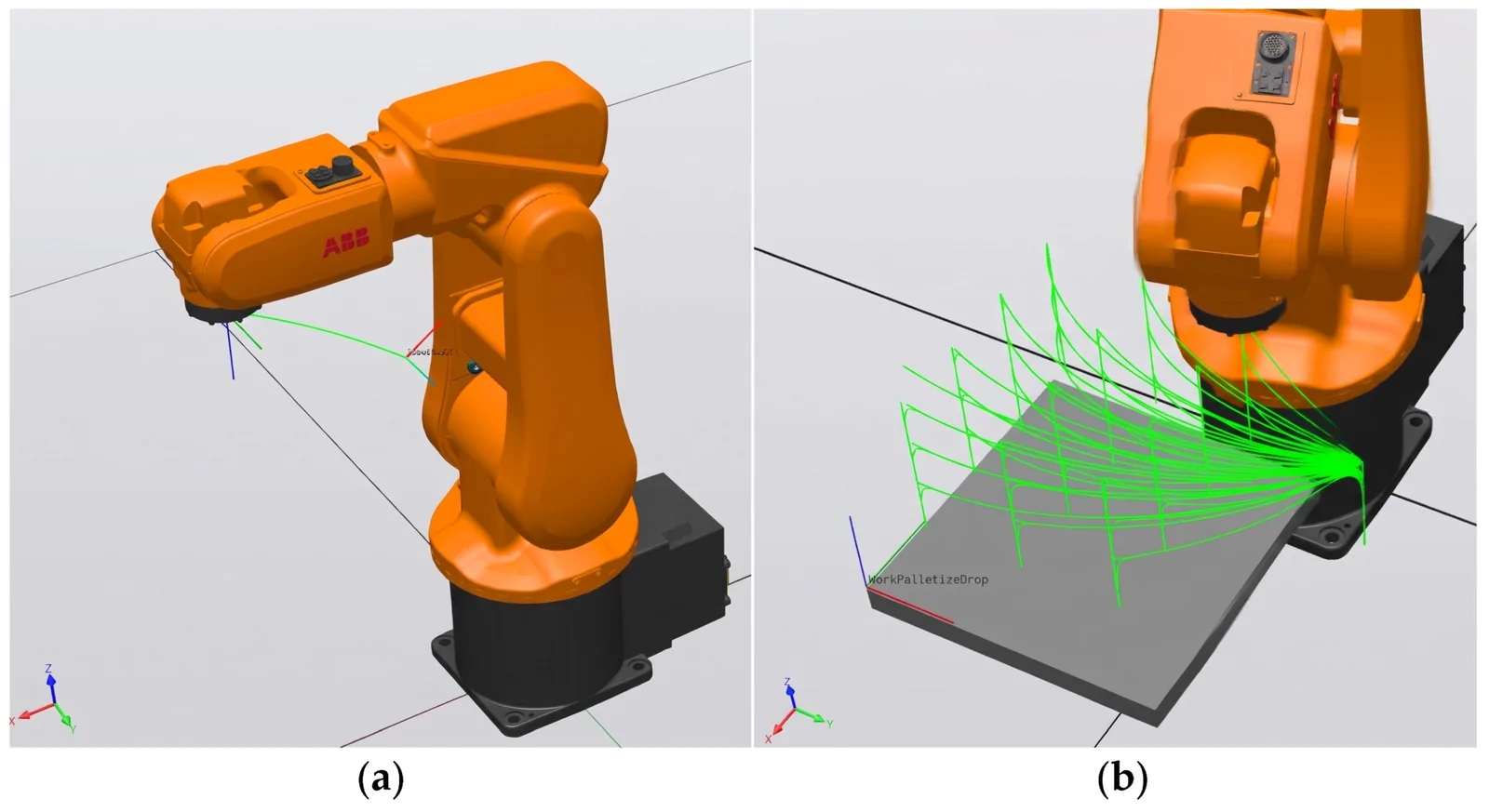
Trajectory of the robot’s movements in the simulation (RobotStudio): (**a**) the robot’s movement to the home position HGR1 (Macro 1); (**b**) the robot’s movement for a specified palletizing process (Prog2 module).

After the connection to the robot is established, a list of available software modules containing control programs (routines) is retrieved from it. The program can be launched by selecting a routine ID from the list or by using one of the three macros (for example, Macro 1 launches the routine that moves the robot to its home position; Figure 17). During testing of the remaining two macros, the robot was moved to its home position (Macro 1), moved along specified trajectories (Macros 2 and 3), stopped, and then resumed operation. Each of the three command sequences was evaluated in 30 trials. Sequences 1 and 2 involved predefined robot program macros, whereas Sequence 3 involved selecting and launching a specific routine from the list of available programs. The results are presented in Table 3.

- Operator Error: The operator errors occurred primarily during longer command sequences and were associated with incorrect recall or execution of the required gesture order.

### 3.4. Robot Motion Test (Robot Guidance)

For safety reasons, directional robot motion tests were performed with the end-effector speed limited to 1 mm/s. The tests were conducted in automatic mode. Motion was generated from commands from a 3-cell memory buffer, which contained three consecutive actions to be performed. Each cell specifies the direction in which the end-effector moves 1 mm in the robot’s reference frame. When movement in a given direction is activated, a new movement instruction is sent to the robot each time the system reads a new gesture label. Changing the gesture that sustains the movement command or terminating the connection causes the end-effector to stop after the queued motion commands have been executed. The test involved commanding a nominal 10 mm end-effector displacement in each of the six Cartesian directions, with 30 repetitions per direction. The test results are presented in Table 4.

Gestures were performed slowly and deliberately during the motion control experiment (Table 4). The mean measured displacement exceeded the nominal 10 mm command in all six directions. The average overshoot was 2.42 mm, corresponding to 24.2% of the commanded displacement. This systematic deviation may be related to the three-element motion command buffer, because queued 1 mm increments can remain pending when the sustaining gesture changes. Further timestamp-based analysis of the command queue is needed to confirm this interpretation.

## 4. Discussion

The results support the central premise of this study; that is, gesture classification alone is insufficient for generating industrial robot commands because the complete perception-to-control chain must also address data consistency, temporal label fluctuations, command context, and execution behavior. After landmark extraction and population-based filtering, 19,250 samples were retained for model development. The MLP classifier operating on the normalized 63-feature representation achieved an approximate mean per-class recall of 99.46%, with class recalls ranging from 98% to 100%. These values indicate that the selected static gestures were highly separable in the held-out test subset. However, because precision, overall accuracy, and macro-F1 were not retained in the exported results, the classification assessment is limited to metrics reconstructed from the row-normalized confusion matrix.

The temporal processing comparison further shows that high static classification performance does not eliminate the need for label-stream stabilization. Raw classifier output, majority voting, three-consecutive-label confirmation, and the proposed cellular-automaton-based stabilizer were evaluated using 30 repetitions per interface action. Three-consecutive-label confirmation and the CA-based method improved command reliability relative to the raw output and produced comparable results across the evaluated actions. The CA processes the 7 most recent labels from a 30-sample buffer and introduces an approximately 300 ms validation delay. This delay represents a trade-off, in that it suppresses short-lived label fluctuations but reduces interface responsiveness. The results therefore support temporal validation as an intermediate layer between perception and command generation, although the present results do not demonstrate an absolute advantage of CA over all simpler filters.

End-to-end testing revealed an important difference between gesture classification performance and complete task performance. Robot-program control succeeded in 78 of 90 trials (86.7%). The 12 unsuccessful trials were classified as operator errors and occurred primarily during longer command sequences, indicating that sequence recall and execution are important sources of error even when individual gestures are recognized accurately. Cartesian direction control succeeded in 175 of 180 trials (97.2%); two failures were associated with loss of the active context and three with operator error. Loss of the active context can occur when the main hand is no longer detected. The two-hand grammar therefore provided effective contextual separation between mode selection and action execution, while the pattern of errors suggests that longer sequences impose greater memory and procedural demands on the operator.

Directional success must be distinguished from metric motion accuracy. Although 175 trials produced motion in the intended direction, the mean measured displacement exceeded the nominal 10 mm command in all six directions. The overshoot ranged from 0.9 mm for −Z motion to 3.4 mm for −Y motion, with a mean of 2.42 mm, or 24.2% of the commanded displacement. A plausible contributor to this systematic error is the three-element motion command FIFO buffer: 1 mm increments already queued when the sustaining gesture changes may still be executed before the stop condition is recognized and processed. The approximately 300 ms CA validation delay, inter-thread communication, and controller-execution latency may further delay stop propagation. As command-level timestamps were not recorded, the individual contributions of stabilization, communication, controller execution, and buffer draining cannot be isolated experimentally. To reduce this overshoot, future implementations should cancel or flush pending motion increments when the sustaining gesture is released or the interaction state changes.

Regarding operational dependability, temporal stabilization must not be interpreted as a certified safety function. The current implementation uses deterministic label and context handling; namely, loss of hand detection produces the operational “None” label, and the CA rules suppress short-lived or ambiguous label changes before they enter the command grammar. Loss of the active command context prevents completion of the intended command sequence. The present study did not quantify communication timeout behavior, stale-command handling, or the priority of explicit stop commands relative to queued motion increments. These behaviors should be verified through controlled interruption tests and timestamp-based queue analysis before deployment in an industrial workcell.

From a workcell integration perspective, the proposed gesture-control framework should be treated strictly as a non-safety-rated auxiliary human–machine interface intended for selected and bounded research tasks. The experimental workstation is part of a laboratory facility and can operate in automatic (AUTO) and manual (MANUAL) modes. The workstation is classified as partly completed machinery and, in its current laboratory configuration, is not equipped with all protective measures required for an independently operated industrial workcell, such as perimeter fencing or safety light curtains. Therefore, the gesture-based interface may be used only under controlled laboratory conditions and does not replace the existing operator controls or safety-related functions of the robot system. Emergency stopping is provided independently of the vision-based interface through the E-STOP devices located on the FlexPendant and the robot controller. These safety devices retain overriding authority over all gesture-generated commands and initiate a Category 0 stop of the robot. Consequently, temporal stabilization and context-dependent command validation should be regarded as mechanisms that improve the operational robustness of the interface, not as certified safety functions. Any deployment outside the laboratory would require a workcell-specific risk assessment, installation of the necessary protective measures, and validation of the complete safety architecture.

The study has several limitations. Validation focused on the algorithmic pipeline and one robotic platform rather than on a multi-operator industrial trial. The experiments did not systematically vary illumination, background, camera distance, or gesture speed, and the vocabulary was limited to 13 active gestures. The selected filtering threshold of −0.25 was evaluated as a compromise between removing inconsistent samples and preserving class coverage, but its transferability to other datasets and vocabularies remains unverified. The study also did not measure the complete gesture-to-command latency, false command rate, rejected valid command rate, workload, or ergonomics. Future work should therefore include multiple operators, controlled environmental conditions, timestamp-based queue latency profiling, usability assessment, controlled communication failure tests, explicit verification of stop-command priority, and evaluation of the interface within a workcell governed by independent certified safety functions.

The threshold sensitivity analysis showed that the selected value of −0.25 represented a dataset-specific compromise among generalization performance, training set coverage, and landmark population consistency. Its applicability to other datasets, gesture vocabularies, and acquisition conditions therefore requires separate validation.

Overall, the results show that a lightweight landmark classifier, temporal stabilization, and context-dependent command mapping can form a practical contactless interface for industrial robot control. The main contribution is not the classifier alone, but the integration of perception, temporal validation, command context, and robot execution. At the same time, the 300 ms validation delay and 24.2% mean displacement overshoot demonstrate that robustness and responsiveness must be optimized jointly before the framework can support applications requiring precise motion commands.

## 5. Conclusions

This article presented a comprehensive algorithmic framework for contactless control of an ABB industrial robot using hand gestures. The framework combines MediaPipe landmark detection, normalization, population filtering, MLP classification, deterministic transition stabilization, and query-based two-handed command mapping. Unlike approaches that directly map labels to actions, the proposed method accepts only stable transitions and then interprets them within the active context of the command.

The principal contribution is treating the HGR results as signals that require verification before controlling an industrial robot. The finite stabilizer suppresses short-duration recognition pulses, and the two-handed grammar separates mode activation from command execution. This is particularly important for industrial robots, where the interaction system must limit unintended movements and remain scalable for various workcell programs.

Future work will expand the framework in four areas. First, the framework should undergo real-world validation involving multiple operators, covering various distances from the camera, backgrounds, lighting levels, and gesture execution speeds. Second, the system should be expanded to include operational safety metrics, such as the percentage of false commands, the percentage of commands skipped, the average activation delay, and the time to return to a safe state. Third, the MLP classifier should be compared with sequential models, such as LSTM or Transformer models, using the same stabilization layer and command grammar. Fourth, the framework should be integrated with the robot’s formal safety constraints, such as work zones, speed limits, stop priority, and verification that commands comply with the robot’s current program state.

## Figures and Tables

**Table 1:** Change in the number of samples relative to the baseline after normalization and filtering using a threshold parameter of −0.25, using a population of 2000 initial samples for each class.

Process	Sample Quantity [pcs]	Relative to the Base [%]	Mean Class [pcs]	Majority Class [pcs]	Minority Class [pcs]
Base	26,000	100	2000	2000	2000
Recognition	29,347	112.9	2257	2541	2105
Filtration	19,250	74.0	1480	1636	1266

**Table 2:** Classification performance of the MLP classifier.

Metric	Value
Number of gesture classes	13
Number of input features	63
Approximate mean recall per class	99.46%
Minimum class recall	98%
Maximum class recall	100%

For a multiclass problem, balanced accuracy corresponds to the unweighted mean of the class-wise recall.

**Table 3:** Robot program control test.

Action	Gesture Sequence	Attempts	Correct Attempts	Operator Error
Sequence 1: Macro 1, Macro 2, STOP, RESUME	CH: call → one → call → peace → grip → dislike → grip → like	30	26	4
Sequence 2: Macro 1, Macro 3, STOP, RESUME	CH: call → one → call → three → grip → dislike → grip → like	30	27	3
Sequence 3: Run a specific module from the accessible program list	CH: fist → rock → one → rock → stop	30	25	5

CH—command hand.

**Table 4:** Robot motion test (robot guidance).

Direction	Gesture Sequence	Mean Measured Displacement [mm]	Mean Absolute Error [mm]	Directionally Successful Trials	Context-Loss Error	Operator Error
−Z	CH: grip → like	10.9	+0.9	30	0	0
+Z	CH: grip → dislike	12.8	+2.8	30	0	0
−X	CH: grip → thumb_index	12.5	+2.5	28	2	0
+X	CH: grip → call	12.7	+2.7	28	0	2
−Y	CH: grip → little finger	13.4	+3.4	29	0	1
+Y	CH: grip → one	12.2	+2.2	30	0	0

CH—command hand.

## Data Availability

The processed landmark dataset, the trained MLP model, the cellular automaton implementation, the command-mapping configuration, and the experimental test results supporting the findings of this study are available: https://github.com/szarbotek/HGR-system-ABB-120 (accessed on 21 September 2026). The source images are available from the original HaGRIDv2 dataset under its applicable license.

## Abbreviations

The following abbreviations are used in this manuscript:

AI	Artificial Intelligence
CART	Classification and Regression Tree
CNN	Convolutional Neural Network
DL	Deep Learning
EMG	Electromyography
GUI	Graphical User Interface
HaGRIDv2	Hand Gesture Recognition Image Dataset, Version 2
HCI	Human–Computer Interaction
HGR	Hand Gesture Recognition
HRI	Human–Robot Interaction
LDA	Linear Discriminant Analysis
ML	Machine Learning
MPR	MediaPipe Recognizer Thread
MTD	Multithreading
RGB	Red-Green-Blue
RGB-D	Red-Green-Blue-Depth
RP	Population Distribution Filter
ReLU	Rectified Linear Unit
sEMG	Surface Electromyography
SHREC	3D Shape Retrieval Contest
SVM	Support Vector Machine
T-SNE	t-Distributed Stochastic Neighbor Embedding
UGV	Unmanned Ground Vehicle
